# Fanconi anemia patients with head and neck squamous cell carcinoma - a multi-center study

**DOI:** 10.1007/s00405-025-09466-x

**Published:** 2025-05-21

**Authors:** Nir Tsur, Anner Moskovitz, Yehuda Zadik, Ehud Even-Or, Narin Nard Carmel Neiderman, Elchanan Zloczower, Omry Frig, Orna Steinberg-Shemer, Hannah Tamary, Noga Kurman, Inna Ospovat, Eyal Yosefof

**Affiliations:** 1 Department of Otolaryngology-Head and Neck Surgery, Rabin Medical Center, Petach Tikva, Israel; 2 Faculty of Medicine, Tel Aviv University, Tel Aviv, Israel; 3 Department of Otolaryngology, Head & Neck Surgery and Maxillofacial Surgery, Tel-Aviv Sourasky Medical Center, Tel-Aviv, Israel; 4 Department of Oral Medicine and Saligman Clinics, Faculty of Dental Medicine, Hebrew University of Jerusalem and Hadassah Medical Center, Jerusalem, Israel; 5 Department of Bone Marrow Transplantation and Cancer Immunotherapy, Hadassah-Hebrew University Medical Center, and Faculty of Medicine, Hebrew University of Jerusalem, Jerusalem, Israel; 6 Department of Hematology-Oncology, Schneider Children’s Medical Center of Israel, Petach Tikva, Israel; 7 Department of Otolaryngology-Head and Neck Surgery, Kaplan Medical Center, Rehovot, Israel; 8 Pediatric Hematology Laboratory, Felsenstein Medical Research Center, Petach Tikva, Israel; 9 Department of Radiotherapy, Davidoff Cancer Center, Rabin Medical Center – Beilinson Hospital, Petach Tikva, Israel; 10 Oncology Division, Tel Aviv Sourasky Medical Center, Tel Aviv, Israel

**Keywords:** Fanconi anemia, HNSCC, Chemoradiation therapy, Oral cancer, Survival

## Abstract

**Objectives:**

To investigate the presentation, treatment, and outcomes of head and neck squamous cell carcinoma (HNSCC) in patients with Fanconi anemia (FA), a rare genetic disorder characterized by increased cancer risk and treatment complications.

**Methods:**

We conducted a retrospective cohort study of 11 FA patients diagnosed with HNSCC across five XXX medical centers from 2014 to 2023. Data on patient demographics, tumor characteristics, treatment modalities, complications, recurrences, and survival outcomes were analyzed using descriptive and survival statistics.

**Results:**

FA patients developed HNSCC at a median age of 31.5 years, primarily in the oral cavity. Surgical treatment was the primary treatment modality; however adjunct radiotherapy resulted in severe complications, such as high-grade mucositis in 75% of cases. Median overall survival was 28.7 months, with 36.4% of patients succumbing within 12 months of diagnosis. Recurrences were noted in three patients, split equally between local and distant sites, and 36% developed secondary malignancies up to 17 years post-initial diagnosis.

**Conclusions:**

HNSCC in FA patients presents distinct challenges, including a younger onset age, high rates of severe treatment complications, and poor survival outcomes. Tailoring treatment strategies to minimize radiotherapy exposure and implementing rigorous, long-term surveillance for second malignancies are essential for managing these high-risk patients.

**Level of Evidence:**

Level IV.

## Introduction

Fanconi anemia (FA) is a rare genetic disorder characterized by impaired correction of DNA damage, leading to a spectrum of clinical manifestations, including bone marrow failures, congenital malformations, and predisposition to malignancies [1]. Specific populations show higher incidences due to founder mutations and consanguinity factors [2–4].FA is caused by mutations in over twenty genes, most inherited in an autosomal recessive pattern. The projected median Survival for FA patients is around 30 years, though this may vary significantly [5]. Cancer, particularly hematologic malignancies like acute myeloid leukemia (AML) and myelodysplastic syndrome (MDS), remains the leading cause of death among Fanconi anemia patients [6, 7]. Many patients with Fanconi anemia undergo allogeneic hematopoietic cell transplantation (HCT), which remains the only curative option for the hematological manifestations of this disease [8, 9]. Allogeneic HCT has shown promising outcomes, with recent studies reporting five-year overall survival rates of 83.2% for pediatric and young adult patients [10]. However, solid tumors are also remarkably prevalent, particularly in younger patients [11, 12]. Unlike MDS and AML, in which the risk peaks between the ages of 30 and 40, [13–15], the risk of developing solid tumors continues to elevate annually. By age 45, about 76% of FA patients are likely to have experienced a solid tumor, with a median age of onset at 28.9 years, compared to 68 years in the general population [6, 16]. This increased incidence includes a significant number of head and neck squamous cell carcinomas (HNSCC), frequently originating in the oral cavity, often without the common risk factors of alcohol or tobacco use found in the general population [13].

Standard treatments for HNSCC typically involve surgery, radiotherapy, chemotherapy, and immunotherapy [17] tailored to the tumor's specific characteristics. However, FA patients often experience severe adverse effects from these treatments, mainly from radiotherapy [11] as well as alkylating agents [17]. High-grade oral mucositis, for instance, affects up to 34% to 65% of the general population undergoing radiotherapy for HNSCC but is reported in up to 75% of FA patients [18, 19]. Complications such as pancytopenia are common in nearly half of the FA patient cohort, compounded by an underlying stem cell disorder that delays the recovery of blood cells, elevating the risk of systemic complications [11]. By the time many FA patients develop head and neck cancer, they have often already undergone allogeneic hematopoietic stem cell transplantation (HSCT) [17]. Recent advances in HSCT for FA patients have improved 5-year survival rates, ranging from 70 to 94% [17]. However, even after successful HSCT, these patients remain at high risk for developing solid tumors, particularly HNSCC, which occurs at a rate 500–800 times higher than in the general population [17].

This multi-institutional study builds upon prior single-site investigations, incorporating a more extensive cohort of FA patients with HNSCC treated across various tertiary medical centers. By analyzing a heterogeneous patient population, this research endeavors to gain deeper insights into FA's distinct genotypic and phenotypic manifestations, thereby providing a more comprehensive understanding of the tumor features, treatment responses, and clinical outcomes in this complex patient demographic. The study is particularly relevant in XXX, where unique genetic populations, including Arabs, Bedouins, and Ashkenazi Jews, exhibit high rates of consanguinity and a narrow genetic load. In XXX, 57% of FA patients are of Arab descent, with 63% being offspring of consanguineous parents [4]. Among Ashkenazi Jews, carrier frequencies for specific FA mutations are high, with 1:92 individuals carrying the FACC IVS4 + 4 A > T mutation [20]. This genetic landscape contributes to a higher incidence of FA and other inherited disorders in these populations, underscoring the importance of comprehensive genetic screening and counseling [21, 22].

## Materials and methods

### Study design

We conducted a retrospective cohort study analyzing data from patients diagnosed with HNSCC and genetic diagnosis confirmed Fanconi anemia. The study encompassed data collected from XXX five largest tertiary otolaryngology centers from January 2014 to December 2023. Each participating center provided comprehensive data on the total number of head and neck cancer patients treated during the study period and detailed records for those also diagnosed with Fanconi anemia.

This study was conducted in accordance with the ethical standards of the institutional and/or national research committee and with the 1964 Helsinki Declaration and its later amendments or comparable ethical standards- IRB RMC 0731–21.

### Data collection

Data were systematically gathered from five large referral centers in XXX, serving approximately 50% of the country's population. All head and Neck patient charts were consequently reviewed, and FA patients were retrieved. We meticulously reviewed medical records to extract relevant information on patient demographics, disease characteristics, treatment modalities, and clinical outcomes. The primary variables collected included demographic data such as age at diagnosis, gender, ethnic origin, smoking, and alcohol consumption background, the genetic cause of Fanconi anemia, previous hematopoietic cell transplantation (HCT), primary tumor site, tumor staging, treatment approaches (including surgery, chemotherapy, and radiotherapy), and any complications, recurrences, and survival rates. We investigated the different complementation groups associated with the disease to analyze the genetic heterogeneity of Fanconi anemia (FA). The study focused primarily on groups A, B, and C, which collectively account for most FA cases [23, 24].

### Statistical analysis

Data was processed and analyzed using the SPSS software package, version 25 (IBM Ltd., Chicago, Il, USA). Survival time was calculated with the difference between the date of death or last follow-up and the date of biopsy or surgery, respectively. The disease-specific survival time was calculated as time elapsed in months between the biopsy or surgery date and the date of last follow-up or death from the disease. Recurrence-free survival time was calculated as time elapsed in months between disease recurrence and surgery. Overall and disease-specific survival times were estimated using the Kaplan–Meier method.

## Results

### Patient characteristics

Between 2014 and 2023, out of a cohort of approximately 5000 patients treated surgically for head and neck cancer at the five participating centers in XXX, 11 patients were diagnosed with FA. The FA patients consisted of 5 males (45.5%), with a median age at the onset of malignancy of 31.5 years (interquartile range [IQR], 29.3–34.3). Five patients had FA group A (45.5%), one had group C (9.1%), and one was a compound heterozygote (9.1%). Notably, seven patients (63.6%) had previously undergone HCT at a median age of 14.7 years (IQR, 7.5–19.5), and two of them suffered from Graft Versus Host disease (GVHD). In addition to FA and HCT, the patient histories revealed varied preconditions: three were smokers (27.3%), two (18.2%) suffered from myelodysplastic syndrome, and three (27.3%) had been previously diagnosed with other solid tumors. One individual developed a second primary head and neck cancer twelve years following their initial treatment.

### Primary tumor characteristics

Analysis of the primary tumor sites showed a predominance in the oral cavity, accounting for five cases (45.5%), followed by the esophagus with four cases (36.4%). Other sites included the larynx, oropharynx, and hypopharynx (9.1% each). Half of the 12 primary tumors presented at stages 3–4 (50%). One had metastatic disease at presentation, three had lymph node metastasis, and four had Very locally advanced primary (cT4). Detailed pathological, surgical, post-surgical, and staging data are presented in Table 1.

**Table 1:** Pathological, treatment, staging details, and oncologic follow-up

Patient num	Primary site	Pathology	Surgical procedure	TNM classification	Stage	Operational complications	Post operational complications	Additional treatment modalities	Treatment complications	Disease recurrence/second primary
1	Esophagus	Untyped	Total esophagectomy	T3 N0M0	2b	No	Post-operational mortality	No		No
2	Esophagus	SCC, with clear cells (positive to cytokeratin 5/6, EMA, BER. Negative to HER2, PAX8)		TxNxM1	4b			Palliative radio-chemotherapy w/CIS 5 FU	Sepsis, Nausea, Fatigue	No
3	Oropharynx	SCC		T2 N0M0	2	No	No	No	No	No
4	Oral	Moderately differentiated SCC	Rt Maxillectomy, unilateral Neck dissection levels 1–3	T4aN0M0	4a	No	No	No	No	No
5	Oral	Moderately differentiated SCC	Subtotal glossectomy, mandibulotomy, and bilateral neck dissection with no complete tumor excision due to intraoperative jugular vein hemorrhage	T4 N2cM0	4a	Bleeding	No	Radiotherapy + Erbitux	No	Distant recurrence in the lung
6	Esophagus	SCC in situ, keratinizing	Total esophagectomy McKeown esophagectomy	TisN0M0	0	No	Dysphagia, weight loss	For the recurrence of Nivolumab	Sepsis	Local recurrence in hypopharynx
7	Larynx	Untyped		Untyped	Untyped	No	No	No	No	No
8	Hypopharynx	Poorly differentiated SCC, keratinizing	Direct laryngoscopy (biopsy)	T4aN0M0	4a	No	No	Adjuvant radio-chemotherapy w/Cis for the recurrence Erbitux	Skin burns, dry mouth	Distant recurrence in the lung
9	Oral	Well-differentiated/Moderately differentiated SCC, ulcerated	Tumor excision with 1 cm margin	T1 N0M0	1	No	No	No	No	Hepatocellular carcinoma
10	Oral	Well-differentiated SCC, keratinizing	Partial glossectomy and bilateral neck dissection	T2 N2cM0	4a	No	No	Adjuvant radiotherapy, for the recurrence Erbitux + CarboTaxol	Mucositis	Local recurrence in the tongue
11	Oral	Undifferentiated SCC	Partial glossectomy and unilateral supra-omohyoid neck dissection	TisN0M0	0	No	No	No	No	Breast, Lung, Skin
11	Esophagus	Moderately differentiated SCC	Total esophagectomy and gastric pull-up	T4 N1M0	3b	Bleeding	No	No	No	

CarboTaxol—carboplatin and paclitaxel; *CIS* cisplatin, *SCC* squamous cell carcinoma, *5 FU* fluorouracil

### Treatment and complications

Treatment modalities included surgery as the cornerstone for most cases (n = 9), supplemented by adjuvant therapies in select cases. Of the four patients who received radiotherapy (27.3%), three received it as an adjuvant to surgery and one as a definitive therapy. The irradiation dose for adjuvant radiotherapy was 59 Gy, 60 Gy, and 65 Gy in 1.8 Gy per fraction, and the patient treated with definitive irradiation received 70 Gy in 2 Gy per fraction. Regarding irradiation methods, one patient received intensity-modulated radiation therapy (IMRT), and two received 3D Conformal radiotherapy (3DCRT). One of these patients also received cetuximab, and another received concurrent chemotherapy with weekly cisplatin (40 mg/m2), which was stopped after 4 out of 7 courses due to toxicity. Two of the patients treated with radiotherapy as an adjuvant to surgery experienced significant treatment toxicity, including dermatitis and xerostomia in one patient and mucositis in another; both were Grade 3 toxicity. Palliative care consisting of concurrent chemoradiotherapy with cisplatin, and fluorouracil was administered to a patient with a history of hematopoietic cell transplantation (HCT) who presented with cervical esophageal squamous cell carcinoma (SCC) accompanied by cervical and mediastinal lymph node metastases as well as liver metastases. The patient experienced significant adverse effects, including fatigue and nausea, and ultimately succumbed to a pulmonary infection and sepsis 4 months after diagnosis. (Table 1).

### Recurrence and second malignancies

The cohort exhibited a notable recurrence rate, with local recurrence occurring in 3 out of 11 patients (27%) and distant recurrence in another three (27%). The time to recurrence varied significantly, ranging from 5 to 50 months, with a median interval of 12 months. Management strategies for recurrent disease varied, encompassing repeated surgeries for two patients, immunotherapy using nivolumab for another two, and a mix of chemotherapy and immunotherapy for one individual. All three patients who received immunotherapy had undergone HCT transplantation previously, and one was also diagnosed with GVHD earlier. Among these, the patient with a prior GVHD diagnosis experienced sepsis resulting from a pulmonary infection, although no further complications from the immunotherapy were noted. In addition, four patients (36%) developed second primary malignancies, including cancers of the breast, hypopharynx, skin, and hepatocellular carcinoma. Notably, one patient with a previous smoking history developed a second primary HNSCC 12 years after their initial cancer diagnosis, which was treated surgically. The time to second primary cancers ranged from 5 to 17 years following the first HNSCC, with a median of 13 years; This data is further described in Table 1.

### Survival and mortality

Among the cohort studied, 10 out of 11 patients (90.9%) succumbed to their conditions, including one individual who passed away from mediastinitis on postoperative day 13 following a total esophagectomy. The mean overall Survival from diagnosis for the remaining ten patients was 44.9 months (IQR, 7.4–51.8), and the median overall Survival was 28.7 months. Four patients (36.4%) died within a year from malignancy diagnosis. The median age at death was 34.9 years (IQR, 30.6–37.7). Causes of death varied, including multi-system failure due to cancer progression (three patients), pneumonia or respiratory failure (two patients), bleeding from the tumor (one patient).

### Longitudinal follow-up

**Fig. 1:**
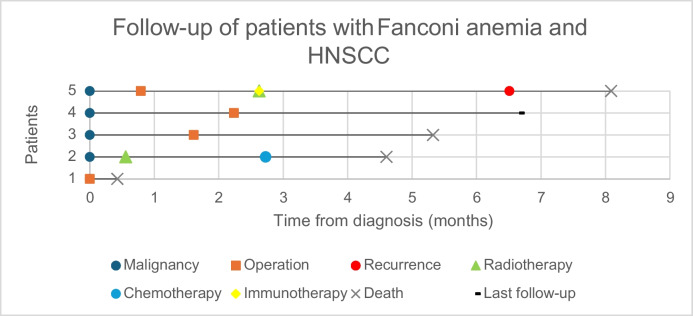
Survival and Treatment Timelines in Patients with Fanconi Anemia and HNSCC with overall Survival of ≤ 1 year from diagnosis. Footnote: Events include malignancy diagnosis, treatments, and outcomes

Long-term follow-up data, illustrating patient outcomes over time, are summarized in Fig. 1, visually representing survival and disease progression across the patient cohort.

## Discussion

**Fig. 2:**
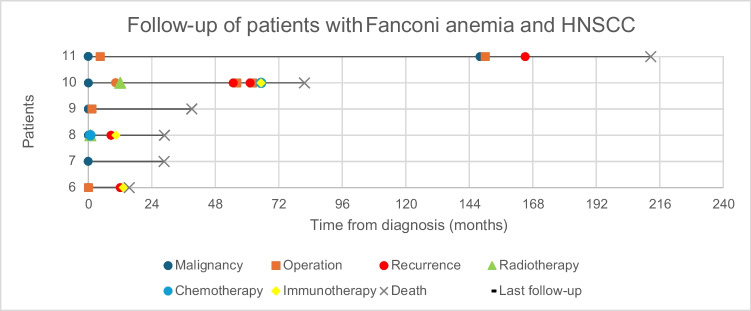
Survival and Treatment Timelines in Patients with Fanconi Anemia and HNSCC with overall Survival of ≥ 1 year from diagnosis. Footnote: Events include malignancy diagnosis, treatments, and outcomes

Our study underscores the distinctive clinical profile and significant challenges faced by patients with Fanconi anemia who develop head and neck squamous cell carcinoma. The key findings reveal that FA patients typically present with HNSCC at a notably younger age, with a median onset of 31.5 years, compared to the general population. Post-treatment complications are a significant concern, with a high incidence of severe adverse effects, particularly from radiotherapy. The short-term mortality is alarmingly high, with a median disease-free survival of just 12 months and a median overall survival of 29 months post-diagnosis. Additionally, there is a significant risk of developing second primary tumors, as observed in one patient developing a second primary HNSCC 12 years post-initial treatment and several others developing malignancies in different sites up to 17 years after the first HNSCC. This further complicates the clinical management and prognosis for these patients (Fig. 2).

### Early age of diagnosis

Our study found that patients with FA typically develop HNSCC at a median age of 31.5 years, which is significantly younger than the general population, where the median age of onset for HNSCC is around 60–70 years [25]. This early onset aligns with previous studies highlighting FA patients'propensity to develop malignancies at a younger age due to their genetic predisposition and defective DNA repair mechanisms [2, 26, 27]. The elevated risk in FA patients underscores the importance of early surveillance and prompt diagnosis.

### Treatment-related complications and mortality

The management of HNSCC in FA patients is particularly challenging due to the severe adverse effects associated with standard treatments. Our findings indicate a high incidence of treatment-related complications, especially from radiotherapy, which resulted in grade III-IV mucositis in 75% of FA patients compared to 34%−57% in the general population [11]. Additionally, one patient experienced grade III-IV skin burns and dry mouth, further highlighting the vulnerability of FA patients to radiation-induced toxicity.

The median overall Survival was 29 months, and the mean overall Survival was 45 months, with 36.4% of the deaths occurring within the first 12 months following diagnosis. This starkly contrasts the general HNSCC population, where median survival rates are generally higher, with studies showing 5-year survival rates of 68.5% overall, 86.6% for localized disease, 69.1% for locally advanced disease, and 39.3% for metastatic disease [28–30].

The increased sensitivity of Fanconi anemia (FA) patients to radiotherapy and chemotherapy, contributing to higher rates of morbidity and mortality, is fundamentally rooted in their inherent DNA repair deficiency [17, 31]. FA is characterized by mutations in at least 22 different genes (FANCA-FANCW) responsible for DNA damage response, specifically impairing the repair of interstrand crosslinks (ICLs) and other critical DNA lesions [31, 32]. During radiotherapy, normal cells can repair DNA damage between treatment fractions, but FA patients'cells lack this crucial repair mechanism [17]. Consequently, each radiation dose compounds unrepaired genetic damage, leading to exponentially increased cellular toxicity [17, 33].This genetic vulnerability means standard therapeutic doses can cause catastrophic cellular destruction, resulting in severe treatment-related complications [17, 33]. Studies have documented that FA patients experience significantly higher rates of treatment-related toxicities, with up to 75% developing severe mucositis and 67% experiencing dysphagia, often preventing completion of planned treatment protocols [11]. In fact, radiotherapy could not be completed in 5 out of 12 cases in one study, with 4 patients dying during the course of radiation [11].The impaired DNA repair pathway thus transforms standard cancer treatments from potentially curative interventions into potentially fatal experiences, dramatically increasing short-term mortality risks [17, 33]. This is exemplified by case reports of young patients without a prior FA diagnosis who developed severe toxicity, including fatal outcomes, from standard cancer treatments [17]. These findings underscore the necessity for highly specialized, carefully modulated therapeutic approaches in FA patients with head and neck squamous cell carcinoma [11, 17, 33].

In comparison to these studies, our cohort confirms the exceptionally high risk of mucositis and other complications in FA patients undergoing radiation treatment. In a study involving FA mice, mucosal ulceration reached 59.4% after 26 Gy of radiation compared to 21.7% in non-FA controls [34]. Similarly, a clinical case of an FA patient undergoing radiotherapy for oropharyngeal carcinoma reported Grade 3 mucositis after just 20 Gy of radiation, requiring a treatment break [35]. The use of IMRT in one of our patients, compared to the two treated with 3DCRT, suggests a potential reduction in treatment-related toxicity, especially mucositis. Recent advances in IMRT have shown promise in minimizing radiation exposure to surrounding healthy tissues, which is particularly beneficial for FA patients with their inherent susceptibility to DNA damage [36]. As more centers adopt IMRT, this modality may offer a safer alternative to 3DRT, potentially reducing the overall radiation toxicity, particularly mucositis, in this vulnerable patient population. However, although IMRT offers superior dose conformity to target volumes, the increased low-dose radiation scatter to larger areas of normal tissue remains a significant concern [17]. These findings underscore the necessity for tailored treatment strategies to mitigate the adverse effects of standard therapies in FA patients while exploring newer technologies to improve outcomes.

### Second primary tumors

FA patients are at a substantial risk of developing second primary tumors. In our cohort, three patients (27.3%) developed a second primary cancer, with one patient developing a second primary HNSCC 12 years after the initial diagnosis, another patient developing malignancies at different sites, including the breast, lung and skin, and a third patient developing hepatocelullar cancer. The time to develop second primary tumors varied widely, emphasizing the need for continuous long-term surveillance. Previous studies have shown similar high incidences of second primary tumors in FA patients, with one study reporting 18 solid tumors among 14 patients (9.7%) out of 145 FA patients [2]. These tumors often affect the head and neck region, with HNSCC being the most common second primary tumor, followed by esophageal, vulvar, and other solid tumors [26].

In other studies, the median time to develop second tumors has been reported to be 13 years after the initial diagnosis [37], which aligns with our findings. The incidence of second primary tumors in FA patients is considerably higher than in the general population due to their underlying genetic instability, with 27.3% of patients in our cohort diagnosed with a second primary solid tumor [38–40]. Treatment and management of these secondary malignancies present additional challenges and often lead to further complications and increased mortality.

### Limitations

This case series has several limitations. First, it is based on a small cohort drawn from the XXX Fanconi registry, limiting its generalizability. Second, the study lacked a prospectively defined design, as participating physicians reported malignancy developments even after the subjects were entered into the registry. Third, while we considered the completeness and accuracy of the data, no formal audits were conducted for the reporting centers, which may affect data reliability. Despite these limitations, this registry allowed us to evaluate HNSCC in a rare population of patients with a genetic disease characterized by chromosomal instability.

### Recommendations for physicians and caregivers

The recently published clinical care guidelines for Fanconi anemia [41]underscore the critical need for personalized, multidisciplinary approaches to managing malignancies in this vulnerable population. These guidelines emphasize early cancer detection, genetic counseling, and customized therapeutic strategies to address the significant susceptibility of FA patients to treatment-related toxicities. In line with these principles, our findings highlight the substantial challenges associated with head and neck squamous cell carcinoma (HNSCC) in FA patients, particularly their heightened sensitivity to radiotherapy and chemotherapy. Severe treatment-related complications, such as grade III-IV mucositis and systemic toxicities, further complicate clinical management.

**Fig. 3:**
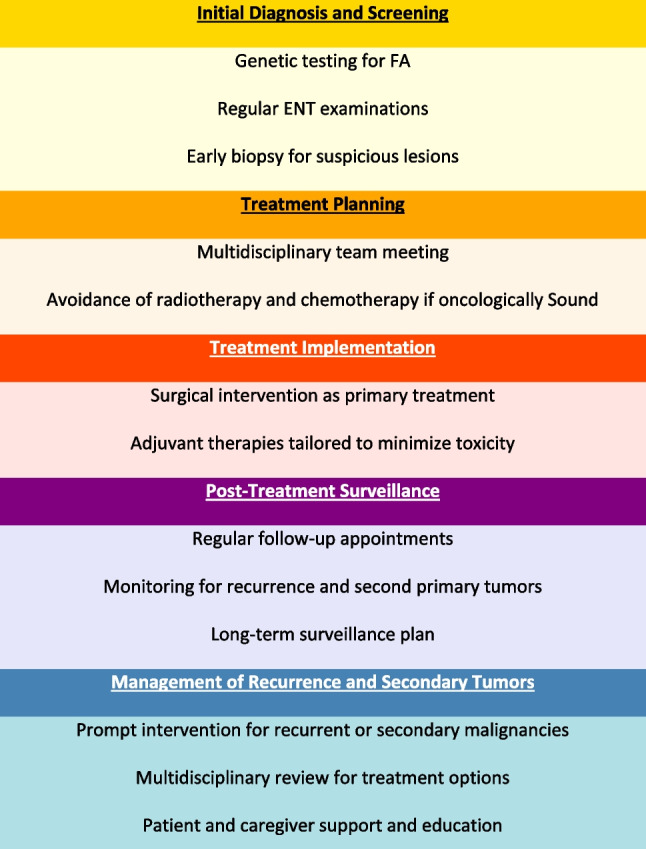
Possible Management of FA Patients with HNSCC

1. Building on our data and these formal guidelines, we propose tailored recommendations for the surgical management of HNSCC in FA patients. These include prioritizing surgery as the primary treatment modality, minimizing the use of radiotherapy and chemotherapy when possible, and employing advanced surgical techniques to achieve optimal oncologic outcomes while reducing toxicity risks. These strategies aim to address the unique challenges of managing HNSCC in this high-risk population:
2. **Early screening and diagnosis:** Initiate routine screening protocols for FA patients starting at age 25, with 6 to 12-month follow-up intervals, to ensure early detection of HNSCC and other malignancies.
3. **Tailored treatment approaches:** Develop and utilize treatment strategies designed explicitly for FA patients to achieve optimal oncologic results while minimizing the use of radiotherapy when possible. Implementing more aggressive and definitive surgical procedures, including free tissue transfer when necessary may make adjuvant chemoradiotherapy unnecessary and enable the Fanconi patient to avoid its possible severe toxicity.
4. **Multidisciplinary care:** Foster a multidisciplinary approach involving oncologists, geneticists, hematologists, ENT and oral medicine specialists, and other specialists to provide comprehensive care for FA patients.
5. **Long-term surveillance:** Ensure continuous and long-term follow-up to monitor developing second primary tumors and manage them promptly.
6. **Patient and caregiver education:** Educate patients and their caregivers about the controlling modifiable risk factors like smoking and alcohol consumption. As well as symptoms of potential malignancies and the importance of early intervention and consistent follow-up (Fig. 3).

## Conclusion

Our research adds to the existing literature by providing comprehensive epidemiological data and emphasizing the critical need for early and vigilant screening for HNSCC in FA patients. It underscores the importance of tailoring treatment strategies to individual patient profiles, focusing on approaches that are less toxic while maintaining robust oncologic outcomes. This study serves as a foundation for future research and supports the development of specialized, patient-centered treatment protocols that can enhance outcomes for similar populations worldwide.

## Data Availability

The data that support the findings of this study are available from the corresponding author upon reasonable request.
